# MicroRNAs and Cardiovascular Disease in Diabetes Mellitus

**DOI:** 10.1155/2017/4080364

**Published:** 2017-02-12

**Authors:** Yue Ding, Xue Sun, Peng-Fei Shan

**Affiliations:** ^1^Department of Endocrinology and Metabolism, The Second Affiliated Hospital Zhejiang University School of Medicine, Hangzhou, Zhejiang, China; ^2^Department of International Health Care Center, The Second Affiliated Hospital Zhejiang University School of Medicine, Hangzhou, Zhejiang, China

## Abstract

Cardiovascular disease (CVD) is the major macrovascular complication of diabetes mellitus. Recently, although CVD morbidity and mortality have decreased as a result of comprehensive control of CVD risk factors, CVD remains the leading cause of death of patients with diabetes in many countries, indicating the potential underlying pathophysiological mechanisms. MicroRNAs are a class of noncoding, single-stranded RNA molecules that are involved in *β*-cell function, insulin secretion, insulin resistance, skeletal muscle, and adipose tissue and which play an important role in glucose homeostasis and the pathogenesis of diabetic complications. Here, we review recent progress in research on microRNAs in endothelial cell and vascular smooth muscle cell dysfunction, macrophage and platelet activation, lipid metabolism abnormality, and cardiomyocyte repolarization in diabetes mellitus. We also review the progress of microRNAs as potential biomarkers and therapeutic targets of CVD in patients with diabetes.

## 1. Introduction

Diabetes mellitus (DM) is a group of chronic metabolic diseases characterized by insulin deficiency and/or insulin resistance that leads to elevated blood glucose levels as well as abnormal fat and protein metabolism [1, 2]. Long-term hyperglycemia can result in both microvascular and macrovascular complications, of which cardiovascular disease (CVD) complications cause the most deaths in patients with diabetes. DM is also an independent risk factor for CVD, excluding other risk factors such as age, hypertension, and obesity [3, 4]. Most patients with CVD often have abnormal glucose metabolism, meaning diabetes and CVD are closely associated. Some studies have proved that high blood glucose levels stimulate the synthesis of advanced glycation end products, advanced oxidation protein products, and oxidation of low-density lipoprotein and that they are related to vascular injury in diabetes through several underlying processes that may be involved in the development and progression of atherosclerosis and could escalate the risk of CVD in patients with diabetes [5].

MicroRNAs are a class of noncoding, single-stranded RNA molecules containing 17–25 nucleotides that posttranscriptionally regulate their target genes by degradation or translational repression of the complementary messenger RNAs (mRNAs) [6]. In this manner, microRNAs modulate several physiological and pathological pathways in human disease, including diabetes, CVDs, cancer, and other diseases. Specifically, several microRNAs are involved in *β*-cell development and function, insulin secretion [7], and insulin resistance in the liver, skeletal muscle, and adipose tissue, which play an important role in glucose homeostasis and the pathogenesis of diabetes [8]. Altered microRNA expression also affects the progression of diabetic complications in the kidney, retina, and peripheral nerves. As each microRNA has the potential to regulate multiple genes in biological processes that include cell proliferation, differentiation, apoptosis, and development, it has been confirmed that the dysregulation of microRNAs affects many pathological pathways in diabetic complications [9].

In the last decade, it has been verified that numerous microRNAs play a pathophysiological role in CVD in diabetes. Both in vivo and in vitro studies have shown that the abnormal expression of microRNAs induced by hyperglycemia causes endothelial cell, vascular smooth muscle cell (VSMC), platelet and macrophage dysfunction, and abnormal lipid metabolism. Clinical studies have indicated that some microRNAs could be diagnostic biomarkers of diabetes and diabetic macrovascular complications. In addition, microRNAs and the genes they regulate could be potential therapeutic targets for CVD in diabetes. In this review, we focus on recent studies in order to elaborate on the role of microRNAs in diabetes-associated CVD based on basic and clinical studies.

## 2. Basic Research on MicroRNAs in CVD in Diabetes

As the main pathological basis of CVD, atherosclerosis is a chronic inflammatory disease of the arteries caused by endothelial cell injury, proliferation of VSMC proliferation, platelet adhesion, and macrophage and lipid accumulation. It has been confirmed that the abnormal expression of microRNAs induced by hyperglycemia is involved in this abnormality. Furthermore, it appears that CVD is aggravated by arrhythmia caused by cardiomyocyte dysfunction, with the involvement of microRNA expression in high-glucose (HG) conditions.

### 2.1. MicroRNAs and Endothelial Dysfunction in Diabetes

**Figure 1:**
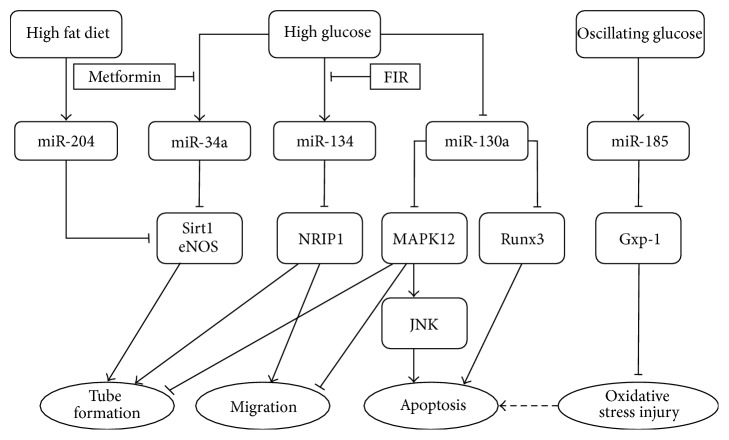
Schematic overview of the role of microRNAs in endothelial cells in hyperglycemia.

Endothelial cells play an important role in the pathological progression of vascular complications resulting from diabetes. Several mechanisms of endothelial dysfunction in DM have been identified, including alteration in signaling related to endothelial nitric oxide synthase (eNOS) activation, increased oxidative stress, activation of the inflammatory processes, and impaired mitochondrial function. Arunachalam et al. [10] found that miR-34a was significantly increased in mouse microvascular endothelial cells in the presence of HG, accompanied by a significant decrease in SIRT1, which deacetylates and activates eNOS and results in impaired angiogenesis. Moreover, treatment with miR-34a inhibitor or metformin downregulated miR-34a expression and upregulated SIRT1 expression, indicating that the hyperglycemia-induced modulation of SIRT1 levels and posttranslational modification of eNOS took place through a miR-34a-dependent gene-regulatory mechanism (Figure 1). Vikram et al. [11] studied the role of miR-204 in endothelial dysfunction in high-fat diet-fed mice. Aortic miR-204 was upregulated, with impaired endothelium-dependent vasorelaxation and increased vascular inflammation. Further research found that miR-204 promotes endothelial dysfunction by targeting* Sirt1* (Figure 1).

Interestingly, a recent study found that miR-185 was upregulated in human umbilical vein endothelial cells (HUVECs) treated with oscillating glucose (OG), while it was unchanged when the cells were treated with HG as compared with cells treated with normal glucose [12]. OG also decreased glutathione peroxidase-1 (GPx-1), which plays a critical role in the enzymatic catabolism of reactive oxygen species (ROS). Cotransfection of HUVECs with the GPx-1  3′ untranslated region (3′ UTR) and miR-185 resulted in significant downregulation of luciferase light emission, indicating that GPx-1 might be a target of miR-185. Therefore, upregulation of miR-185 induced by OG targets GPx-1 to impair the antioxidant response and then caused endothelial cell injury (Figure 1).

Wang et al. [13] investigated the role of miR-134 in endothelial colony-forming cells (ECFCs) from patients with DM and from disease-free (DF) donors (dfECFCs). ECFCs from the patients expressed higher levels of miR-134 than that from the DF subjects and showed reduced cell migration and formation of microvasculature structure. dfECFCs treated with HG to mimic hyperglycemia or with HG combined with low growth factor (LGF) conditions (HG/LGF) to mimic the poorest progression of patients with DM showed decreased cell mobility and tube formation ability, respectively. Subsequently, miR-134 overexpression in dfECFCs contributed to a 3.6-fold increase in miR-134 and reduced cell migration and tube formation ability. Similar findings were also found in HUVECs. Evidence from subsequent research showed that far-infrared radiation (FIR) treatment might suppress miR-134 expression and rescue ECFC function by at least 1.8- and 1.6-fold in terms of cell migration and tube formation ability, respectively. In addition, the* NRIP1* gene, which is involved in diabetes or angiogenesis, is believed to be a target of miR-134, where its expression is reduced in dfECFCs overexpressing miR-134 and is increased in ECFCs treated with FIR. Therefore, the increased miR-134 in HG conditions results in decreased NRIP1 levels, thereby impairing the angiogenic activities of ECFCs, which FIR treatment can reverse (Figure 1).

Xu et al. [14] examined microRNA expression in endothelial progenitor cells (EPCs), which play an important role in vascular repair. miR-130a was significantly downregulated in EPCs from the peripheral blood of patients with diabetes as compared with that of healthy individuals. miR-130a inhibition decreased EPC proliferation, migration, and colony formation but increased EPC apoptosis by targeting RUNX3. Downregulated miR-130a in EPC from patients with DM also dysregulated autophagy and autophagosome accumulation, an effect mediated by RUNX3, which could contribute to excessive autophagic cell death and impaired EPC function. Another study found that miR-130a inhibits the JNK pathway by targeting MAP3K12, contributing to its antiapoptotic effect and the maintenance of EPC function [15]. In diabetic EPCs, HG affects the expression of miR-130a, inducing sustained JNK activation and promoting EPC apoptosis and dysfunction. Consequently, the downregulation of miR-130a might underlie endothelial dysfunction in diabetes through activation of the JNK signaling pathway (Figure 1).

### 2.2. MicroRNAs and VSMC Dysfunction in Diabetes

The majority of cells in the tunica media of the arteries are composed of VSMCs, which are one of the most plastic cells in the human body. In hyperglycemic conditions, VSMCs change their phenotype from a contractile state to synthetic state, representing excessive proliferation, migration, and extracellular matrix secretion, which is believed to contribute to a series of pathological processes relevant to CVD [16]. Recent studies have proven that the modulatory mechanisms of HG-induced VSMC dysfunction are related to microRNA expression.

**Figure 2:**
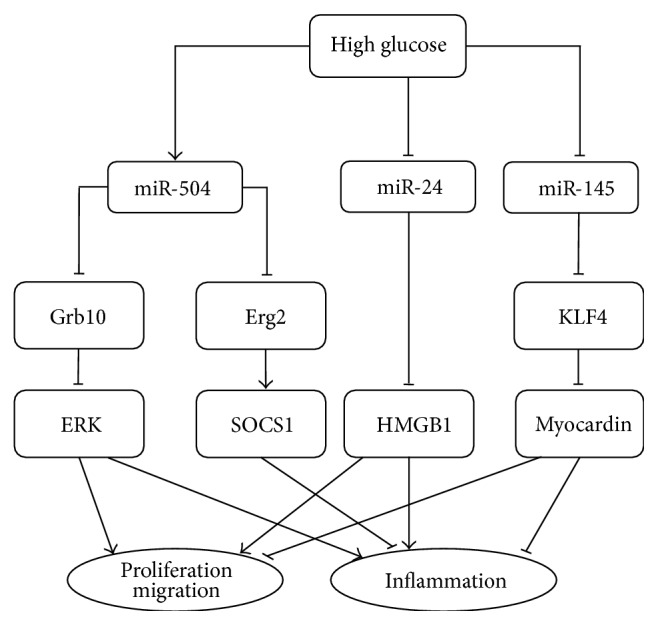
Schematic overview of the role of microRNAs in vascular smooth muscle cell in hyperglycemia or diabetes.

miR-504 levels were significantly upregulated in the aortic VSMCs of diabetic mice; miR-504 promotes VSMC proliferation and migration [17]. The coding gene for miR-504 is in the third intron of the* Fgf13* gene on the X chromosome. In addition, the study found that miR-504 targeted the* Grb10* and* Egr2* genes. In VSMCs, miR-504 overexpression downregulated the expression of* Grb10*, which enhanced ERK1/2 activation, thereby promoting the synthetic phenotype and increasing proinflammatory gene expression, proliferation, and migration. Modulated by miR-504 overexpression or* Grb10* gene silencing,* Egr2* downregulation reduced the anti-inflammatory* Socs1* and increased the expression of several proinflammatory genes, including* Il6*,* Ccl2*, and cyclooxygenase 2* (Ptgs2)* (Figure 2). Therefore, the overexpression of miR-504 in diabetic mice might cause VSMC dysfunction by inhibiting the contractile genes and augmenting the inflammatory genes and proliferation and migration by downregulating* Grb10* and* Egr2* expression [17].

miR-24 is emerging as a regulator of VSMC pathology, targeting genes involved in HG-induced cell proliferation and migration. miR-24 expression was lost in HG-incubated VSMCs in vitro, which corresponded to an increase in HMGB1, a nuclear protein playing an important role in VSMC abnormal proliferation and migration [18] (Figure 2). Transfecting miR-24 into VSMCs markedly upregulated miR-24 expression and inhibited HG-induced VSMC proliferation and migration. As a downstream signaling molecule of HMGB1, NF-*κ*B plays a key role in the inflammatory processes. miR-24 overexpression inhibited the HG-induced activation of NF-*κ*B by suppressing NF-*κ*B p65 translocation and NF-*κ*B DNA-binding activity. Accordingly, miR-24 overexpression significantly decreased the HG-induced secretion of proinflammatory cytokines, including TNF-*α* and IL-6 [18].

Several studies have indicated the critical role of miR-145 in VSMC phenotype switching (Figure 2). Decreasing miR-145 increased the expression of its target gene* Klf4*. It increased* Klf4* and then decreased myocardin to induce VSMC proliferation and migration [19]. A recent study found that miR-145 expression differed according to glucose concentration and duration of glucose treatment [20]. miR-145 was decreased significantly by 12.5–75 mmol/L glucose, with 25 mmol/L glucose (HG) having the maximal effect. Subsequently, 25 mmol/L glucose decreased miR-145 expression maximally at 0.5 h; after 4–6 h, miR-145 expression decreased gradually and was significantly lower than the control level [20]. These results indicate that sustained HG conditions decrease miR-145 expression in VSMCs. HG incubation increased angiotensin II (Ang II) secretion in VSMCs, which inhibited miR-145 expression under HG conditions. Furthermore, the addition of enalaprilat dihydrate and valsartan, an Ang II receptor antagonist, significantly upregulated miR-145 expression under HG stimulation [20].

### 2.3. MicroRNAs and Platelet Activation in Diabetes

Platelet activation plays a key role in the occurrence and development of CVD. Platelet activation, including adhesion, deformation, aggregation, and release, occurs when the vascular endothelium is injured or undergoes certain physiological and pathological stimuli, and this is an important initiating factor in thrombosis. The level of platelet miR-223 was attenuated in patients with diabetes and in mice, which affected platelet function [21]. Platelets from miR-223 knockout mice had greater aggregation and thrombi formation ability, along with longer clot retraction time as compared with platelets from the wild-type littermates [21]. In mice, miR-223 deletion resulted in the increased expression of proteins, such as *β*1 integrin, kindlin-3, and coagulation factor XIII-A (FXIII-A), which are elevated in individuals with diabetes as well. These proteins lead to a hyperreactive and hyperadhesive platelet phenotype. The altered miR-233 levels might have been related to the DM-induced activation of calpain. Calpain is a Ca^2+^-activated cysteine protease that can lead to the proteolytic cleavage of Dicer, an RNAse III, to convert precursor microRNAs into mature microRNAs [22]. Treating diabetic mice with a calpain inhibitor significantly increased the level of platelet Dicer, as well as the expression of miR-233 and its target proteins, that is, kindlin-3 and FXIII-A. These results suggest that the loss of miR-233 enhances platelet reactivity in DM, which is modulated by the activation of calpain [21].

### 2.4. MicroRNAs and Macrophages in Diabetes

Macrophages are the main component of white blood cells in atherosclerotic plaques and are also the main cause of atherosclerotic plaque formation. Macrophages can be divided into M1 and M2 types. The former promotes inflammation and inhibits cell proliferation; the latter promotes cell proliferation and tissue repair [23]. The proportion of the two macrophage phenotypes affects the outcome of atherosclerosis [24]. Sun et al. [25] observed decreased expression of miR-181b in endothelial cells from the epididymal white adipose tissue (eWAT) of insulin-resistant mice and in HG-treated endothelial cells. Next, they demonstrated that the delivery of miR-181b could shift macrophage polarization toward the M2 anti-inflammatory phenotype and reduce macrophage accumulation in eWAT by targeting PHLPP2, which directly dephosphorylates and inactivates AKT at Ser473. However, miR-181b did not inhibit macrophage migratory and proliferative ability [25]. Therefore, decreased miR-181b in insulin-resistant mice increases vascular inflammation and accelerates atherosclerosis.

### 2.5. MicroRNAs and Lipid Metabolism in Diabetes

**Figure 3:**
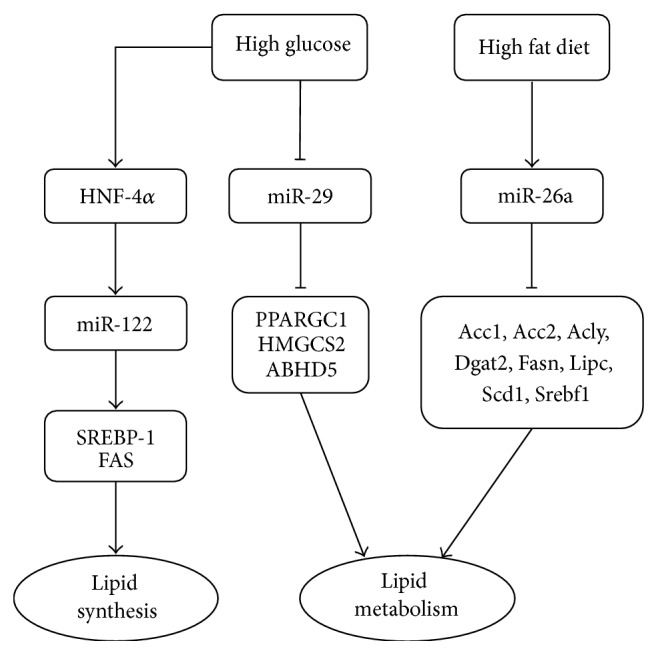
MicroRNAs involved in lipid metabolism.

Abnormality of lipid metabolism is another important risk factor for CVD in patients with DM. Several microRNAs are involved in lipid metabolism, mainly by regulating the expression of genes related to lipid synthesis, transport, and oxidation. The expression of hepatic miR-29 was upregulated in Zucker diabetic fatty* (fa*/*fa)* rats, and miR-29 was an inhibitor of FOXA2-mediated activation of key lipid metabolism genes, including* Ppargc1a*,* Hmgcs2*, and* Abhd5* [26] (Figure 3). Moreover, FOXA2 partly regulated hepatic miR-29 expression. FOXA2 activity was upregulated in insulin-resistant mice, which in turn elevated miR-29 levels [26]. This means that there is a miR-29 regulatory circuit in the liver and that miR-29 is an important regulatory factor in lipid metabolism.

Wei et al. [27] examined the role of miR-122 in regulating lipid metabolism. They found that miR-122 and hepatocyte nuclear factor-4*α* (HNF-4*α*) expression were increased in diabetic mice and in palmitate-treated HepG2 cells. HNF-4*α* is a nuclear receptor protein involved in liver development that modulates miR-122 levels in mouse liver [28]. HNF-4*α* and miR-122 expression in HepG2 cells upregulated the expression of SREBP-1 and FAS, which control cellular cholesterol homeostasis, and activated the genes that control fatty acid, cholesterol, and triglyceride synthesis [27] (Figure 3).

Another study demonstrated that miR-26a was downregulated in obese mouse models as compared with control animals [29]. The overexpression of miR-26a in mice fed a high-fat diet improved insulin sensitivity, decreased hepatic glucose production, and decreased fatty acid synthesis, thereby preventing obesity-induced metabolic complications. Conversely, silencing endogenous miR-26a in conventional diet-fed mice impaired insulin sensitivity, enhanced glucose production, and increased fatty acid synthesis. miR-26a targeted several key genes in lipid metabolism, including* Acc1*,* Acc2*,* Acly*,* Dgat2*,* Fasn*,* Lipc*,* Scd1*, and* Srebf1* (Figure 3).

### 2.6. MicroRNAs and Cardiomyocytes in Diabetes

Arrhythmia is characterized by abnormalities of cardiac origin, heart rate, and rhythm and impulse conduction. Dysfunction of the cell membrane ion channels is an important structural basis of arrhythmia. Li et al. [30] demonstrated that hyperglycemia led to electrophysiological change in cardiac progenitor cells (CPCs), which play an important role in the repair and regeneration of cardiovascular tissues. Furthermore, HG exposure induced augmented miR-1/133 expression in the CPCs. The overexpression of miR-1/133 suppressed KCNE1 and KCNQ1, which both encode the slow delayed rectifier potassium current (*I*_Ks_), playing a key role in restoring the functional *I*_Ks_, which is reduced by the inhibition of KCNE1 and KCNQ1 in diabetic conditions [30].

These studies indicate that microRNAs are differentially expressed and potentially have a pathogenic effect in diabetic CVD. Although many microRNAs have been identified, the mechanisms of their abnormal expression in DM and their target genes required further research and they should be fully characterized. These studies also showed that overexpression or inhibition of certain microRNAs could reverse its pathogenicity, which provides novel therapeutic approaches for alleviating diabetes-induced progression of cardiovascular complications.

## 3. Clinical Studies on MicroRNAs in CVD in Diabetes

In the previous sections, we reviewed the potential molecular pathology mechanisms of microRNAs in CVD in cytological and animal studies. These mechanisms indicate that microRNAs play a crucial role in the pathogenesis of atherosclerosis, which impedes the blood flow of coronary vasculature, followed by the occurrence of ischemic heart disease. Furthermore, epidemiological studies and clinical research on humans have demonstrated the involvement of microRNAs in CVD pathogenesis in diabetes.

### 3.1. MicroRNAs and Risk of Diabetes

A prospective population-based cohort including 80 patients with type 2 DM (T2DM) and 80 age- and sex-matched controls showed that, in patients with diabetes, miR-28-3p was overexpressed, and 12 other microRNAs were underexpressed [31]. Decreased circulating miR-126 was a significant predictor of DM. miR-15a, miR-29b, miR-126, and miR-223 were decreased in the subjects with DM [31]. In pancreatic *β*-cell, islets, enriched miR-375 was increased in subjects with T2DM and modulated *β*-cell function through several physiological mechanisms. miR-375 inhibits insulin secretion and transcription, maintains *β*-cell mass, proliferation, and regeneration, and promotes embryonic pancreas development [32]. Besides, it was found that microRNAs control the insulin signal transduction pathways in target tissues. Insulin resistance refers to the failure of target tissues, including the liver, skeletal muscle, and adipose tissues, to respond adequately to circulating insulin. Clinical studies have reported underexpressed miR-133 [33] and overexpressed miR-503 [34] in skeletal muscle, while increased miR-181a and decreased miR-17-5p, miR-132, and miR-134 have been observed in the omentum. In addition, miR-147 and miR-197 were increased in subcutaneous fat tissue while miR-27a, miR-30e, miR-155, miR-210, and miR-140 were decreased [35]. As the above findings suggest, microRNAs aid in the prognosis of diabetes and could be pharmacological targets in diabetes.

### 3.2. MicroRNAs and Risk of CVD

miR-126 is a human microRNA encoded in the intron of* Egfl7* that controls angiogenesis upon its various transcripts [36]. It is expressed more abundantly than other microRNAs in endothelial apoptotic bodies [37]. miR-126 mediates chemokine factor CXCL12 production, and shedding of miR-126 regulates vascular endothelial growth factor (VEGF) responsiveness and confers vascular protection [38]. The monocytes of patients with DM show impaired responsiveness to VEGF, which might be attributed to the reduced delivery of miR-126 to the monocytes. Recently, it was suggested that miR-126 could be a biomarker of coronary heart disease in patients with T2DM [38]. Compared with healthy controls, the expression levels of circulating miR-126 were decreased in the peripheral blood of patients with T2DM and in patients with T2DM with coronary artery disease (CAD). It has also been suggested that miR-126 correlates negatively with LDL in patients with CAD [39].

Polymorphisms in the corresponding sequence space in the form of single-nucleotide polymorphisms (SNPs) or mutations contribute significantly to phenotypic variation. miR-196a2 is a microRNA encoded by the MIR196A2 gene in humans. It belongs to the miR-196 precursor family, and miR-196a2 T/C polymorphism (rs11614913) is related to thrombosis and inflammation by regulating annexin A1 (ANXA1) in the circulation system [40]. Recently, a prospective case-controlled study of a Chinese population reported that the rs11614913 T → C variation in hsa-miR-196a2 is associated with poor prognosis of CAD [41]. Another study of a Chinese population found that a functional variant of miR-196a2 contributed to susceptibility to congenital heart disease [42]. Furthermore, common genetic polymorphisms in pre-microRNAs are associated with increased risk for dilated cardiomyopathy [43]. Regarding atrial fibrillation (AF), a study involving 123 participants showed that patients with AF with the TC + CC genotype had greater left atrial dimension than patients with the TT genotype, which supports the premise that the pre-miR-196a2 polymorphism is associated with AF and that the C allele is a risk factor for AF [44].

### 3.3. MicroRNAs and Risk Factors and Outcome of CVD

Genetic variants in microRNA genes or the 3′ UTR of microRNA target genes influence microRNA-mediated regulation of gene expression. In this manner, microRNAs influence the susceptibility and prognosis of human diseases. A study of 1004 hospitalized patients in China investigated the effect of microRNA-related polymorphisms on the prognosis of patients with angiographic CAD. The authors found that miR-4513 rs2168518 was associated with blood pressure, lipids, and blood glucose levels, and, as expected, risk for DM. miR-499 rs3746444 and miR-423 rs6505162 were associated with blood pressure and high-density lipoprotein (HDL) levels. Event-free survival was apparently correlated with miR-4513 rs2168518 and miR-499 rs3746444. Furthermore, miR-4513 rs2168518 was associated with increased mortality in patients with CAD. Accordingly, miR-4513 rs2168518 and miR-499 rs3746444 might be biomarkers of the clinical prognosis of CAD [45]. In this case, the findings support the premise that microRNA-related polymorphisms influence clinical outcomes in CAD.

### 3.4. MicroRNAs and Pharmacotherapy of CVD

In the above sections, we reviewed studies clarifying how microRNAs mediate endothelial dysfunction in T2DM. It has been confirmed that this process is associated with the progression of atherosclerosis and neointimal proliferation in patients with T2DM after coronary stenting [46–48]. This emphasizes the importance of molecular targets in drug therapy for improving the endothelial dysfunction of such patients. A previous study observed early decreases in smooth muscle cell migration and proliferation when patients with T2DM were treated with pioglitazone [49]. A subsequent prospective study showed that pioglitazone significantly decreased neointimal hyperplasia (NIH), accompanied by increases in circulating miR-24. We can infer that decreased miR-24 might be associated with increased NIH in patients with T2DM [50].

In the clinic, there is interindividual heterogeneity of platelet response to clopidogrel. Clopidogrel is an irreversible P2Y12 receptor inhibitor. It mediates platelet glycoprotein IIb/IIIa (GPIIb/IIIa) inhibition by inhibiting adenosine diphosphate- (ADP-) induced P2Y12 activation of the downstream pathways and therefore influences vasodilator-stimulated phosphoprotein (VASP) phosphorylation. A recent study confirmed the existence of a microRNA pathway in anucleate platelets in humans and suggested that miR-223 regulates P2Y12 receptor expression [51]. It has been confirmed that decreased platelet miR-223 expression is associated with blunted platelet response to clopidogrel in patients with CHD [52]. In addition, a study of patients with troponin-negative non-ST elevation acute coronary syndrome showed that miR-223 levels correlated negatively with the platelet reactivity index (PRI). The results suggest that circulating miR-223 might be a novel biomarker for assessing the responsiveness to clopidogrel in such patients [53].

With solid evidence of the effects of microRNAs on the CVD pathophysiology increasing and the exciting development of potent microRNAs modulating technologies, microRNAs and the genes they regulate could be potential therapeutic targets for CVD in diabetes. Certainly, several challenges in the form of clinical complication remain. First, individual microRNAs should not be interpreted in isolation because microRNAs may work together, differentially, or in overlapping fashion. The challenge of establishing the role of these microRNAs in diabetic complications and identifying the regulatory mechanisms or pathways related to microRNAs expression in the pathogenesis of the various disorders remains. Second, more studies are needed to develop microRNA therapeutic methods with long half-life and tissue-specific to effectively deliver the microRNAs or their inhibitors to the cardiovascular system. In addition, the effectiveness and safety of long-term microRNAs overexpression or silencing in the clinic are unknown and require more intensive investigation.

Collectively, clinical studies on humans have demonstrated that microRNAs are related to the risk of CVD and could be potential biomarkers of CVD prognosis. Moreover, it has been suggested that microRNAs are correlated to the risk factors of CVD, such as blood pressure, blood glucose, and lipids. In terms of drug therapies, the varied responses correlated to microRNAs imply their potential clinical influence on individualized treatment.

## 4. Conclusions

In this article, we reviewed studies on how microRNAs are involved in CVDs through endothelial dysfunction, VSMC and cardiomyocyte dysfunction, platelet activation, macrophage phenotype, and lipid metabolism in diabetes. Despite the progress in lifestyle management and drug therapy, CVDs remain the most life-threatening complication of diabetes. This emphasizes the need for the integration of molecular research into the diagnosis and treatment of diabetic cardiovascular complications. Studies have explored the possible mechanisms in which microRNAs correlate with coronary heart disease, hypertrophic cardiomyopathy, and arrhythmias. Increasingly, studies have focused on how microRNAs modulate the function of endothelial cells, mast cells, and lipid metabolism. However, as studies among the diabetic population are limited, concrete mechanisms of how a particular microRNA affects different cell types and different cardiac diseases remain unclear. Furthermore, there is limited understanding of the cross-correlation of how different microRNAs act to date. The lack of clinical application also cannot be ignored. We anticipate further understanding of the pathophysiological mechanism in disease development and of the conversion of research findings to realistic predictions of cardiovascular risk and effective management.
